# Prognostic and Clinicopathological Significance of PD-L1 in Patients With Bladder Cancer: A Meta-Analysis

**DOI:** 10.3389/fphar.2019.00962

**Published:** 2019-08-30

**Authors:** Lei Zhu, Jin Sun, Ling Wang, Zhigang Li, Lei Wang, Zhibin Li

**Affiliations:** ^1^Department of Urology, First People’s Hospital of Shangqiu City, Shangqiu, China; ^2^Department of Obstetrics and Gynecology, The General Hospital of Western Theater Command, Chengdu, China; ^3^Department of Urology, Panzhihua Central Hospital, Panzhihua, China; ^4^Department of Urology, The General Hospital of China National Petroleum Corporation in Jilin, Jilin, China; ^5^Department of Urology, Shanxi Provincial Cancer Hospital, Taiyuan, China

**Keywords:** meta-analysis, prognosis, PD-L1, bladder cancer, survival

## Abstract

**Background:** The prognostic role of programmed cell death-ligand 1 (PD-L1) in bladder cancer has been investigated in previous studies, but the results remain inconclusive. Therefore, we carried out a meta-analysis to evaluate the prognostic significance of PD-L1 in patients with bladder cancer.

**Methods:** The electronic databases PubMed, Embase, Web of Science, and Cochrane Library were searched. The association between PD-L1 expression and survival outcomes and clinicopathological factors was analyzed by hazard ratios (HRs) or odds ratios (ORs) and 95% confidence intervals (CIs).

**Results:** A total of 11 studies containing 1,697 patients were included in the meta-analysis. High PD-L1 expression was associated with poor overall survival (OS) (HR = 1.83, 95% CI = 1.24–2.71, *p* = 0.002). There was nonsignificant association between PD-L1 and recurrence-free survival (RFS) (HR = 1.43, 95% CI = 0.89–2.29, *p* = 0.134), cancer-specific survival (CSS) (HR = 1.51, 95% CI = 0.80–2.87, *p* = 0.203), or disease-free survival (DFS) (HR = 1.53, 95% CI = 0.88–2.65, *p* = 0.13). Furthermore, high PD-L1 was significantly correlated with higher tumor stage (OR = 3.9, 95% CI = 2.71–5.61, *p* < 0.001) and distant metastasis (OR = 2.5, 95% CI = 1.22–5.1, *p* = 0.012), while PD-L1 overexpression was not correlated with sex, tumor grade, lymph node status, and multifocality.

**Conclusions:** The meta-analysis suggested that PD-L1 overexpression could predict worse survival outcomes in bladder cancer. High PD-L1 expression may act as a potential prognostic marker for patients with bladder cancer.

## Introduction

Bladder cancer is the most common malignancy of the urinary tract, accounting for 80,470 new cases and 17,670 deaths in 2019 alone in the United States (Siegel et al., 2019). When diagnosed, up to 75% of patients present with non-muscle-invasive bladder cancer (NMIBC), about 20% present with muscle-invasive bladder cancer (MIBC), and 5% would have metastatic disease. Although patients with NMIBC have a relatively good prognosis, the prognosis of regional and distant metastatic disease is poor, with 5-year survival rates of 35% and 5%, respectively (National Cancer Institute SEER Program). Therefore, investigation of novel biomarkers to stratify patients is important for clinical management (Slovin, 2017).

Cancer immunoediting is a process consisting of immunosurveillance and tumor development (Mittal et al., 2014). Programmed cell death-1 (PD-1) and its ligand programmed cell death-ligand 1 (PD-L1) have an important role in the regulation of responses of our immune system (Errico, 2015). PD-L1 is also known as B7-H1, CD274, which is expressed on many cancer cells. PD-L1 expression has shown prognostic value in various tumors including pancreatic cancer (Gao et al., 2018), colorectal cancer (Shen et al., 2019), and non-small cell lung cancer (Ma et al., 2018). Recently, many studies (Nakanishi et al., 2007; Boorjian et al., 2008; Wang et al., 2009; Xylinas et al., 2014; Bellmunt et al., 2015; Wu et al., 2016; Noro et al., 2017; Li et al., 2018b; Pichler et al., 2018; Owyong et al., 2019; Wang et al., 2019) also investigated the prognostic significance of PD-L1 expression in bladder cancer, but the results remain controversial. Therefore, we collected relevant data and performed a meta-analysis to quantify the prognostic role of PD-L1 and analyze the relationship of PD-L1 and clinicopathological parameters in bladder cancer.

## Methods

### Literature Search

This meta-analysis was conducted in accordance with the Preferred Reporting Items for Systematic Reviews and Meta-Analyses (PRISMA) guidelines (Moher et al., 2009). The research of PubMed, Embase, Web of Science, and Cochrane Library identified relevant studies published in English. The last search was updated on March 2019. A comprehensive search strategy was performed based on the following terms: “programmed death ligand-1,” “PD-L1,” “B7-H1,” “CD274,” “bladder cancer,” “bladder neoplasm,” “bladder tumor,” and “bladder urothelial carcinoma.” The references of the included studies were also manually checked to identify relevant publications. Ethical approval was waived because we just collected the data from available publications.

### Eligibility Criteria

The inclusion criteria were as follows: 1) patients were histologically diagnosed to have bladder cancer; 2) PD-L1 was detected *via* immunohistochemical staining (IHC); 3) the relationship between PD-L1 and survival of bladder cancer was studied; and 4) references are published in English. Exclusion criteria were as follows: 1) duplicate studies; 2) studies provided incomplete data; and 3) meeting abstracts, case reports, reviews, or animal studies.

### Data Extraction and Quality Assessment

Two independent investigators extracted the following information from the eligible studies: first author, publication year, country, detection method, sample size, study design, survival analysis, age, and study period. Any disagreement was resolved by discussion. The quality of the selected articles was assessed according to the Newcastle-Ottawa Scale (NOS) (Wells et al., 2009). Total quality score of NOS was ranged from 0 to 9, and studies that scored ≥6 were considered as high-quality studies.

### Statistical Analysis

Hazard ratios (HRs) and their 95% confidence intervals (CIs) were searched in the original articles or calculated by methods described by Tierney et al. (2007). The survival outcomes included overall survival (OS), recurrence-free survival (RFS), cancer-specific survival (CSS), and disease-free survival (DFS). The logHR and standard error (SE) were used to present the survival results. An observed HR > 1 implied a poorer prognosis in patients with high PD-L1 expression, while HR < 1 indicated a better prognosis. The relationship between PD-L1 expression and clinicopathological features was evaluated by odds ratios (ORs) and corresponding 95% CIs. Cochran’s *Q* test and Higgins *I*-squared statistic (*I* ^2^) were used to measure the heterogeneity of the combined HRs (Higgins and Thompson, 2002). *I* ^2^ > 50% and/or *p* < 0.1 suggested significant heterogeneity in terms of statistics, and a random-effects model was utilized. Alternatively, a fixed-effects model was applied. Begg’s test was used to detect potential publication bias (Begg and Mazumdar, 1994). All statistical analyses were conducted by using Stata version 12.0 (Stata Corporation, College Station, TX, USA). A two-sided *p* < 0.05 was considered statistically significant.

## Results

### Study Selection

**Figure 1:**
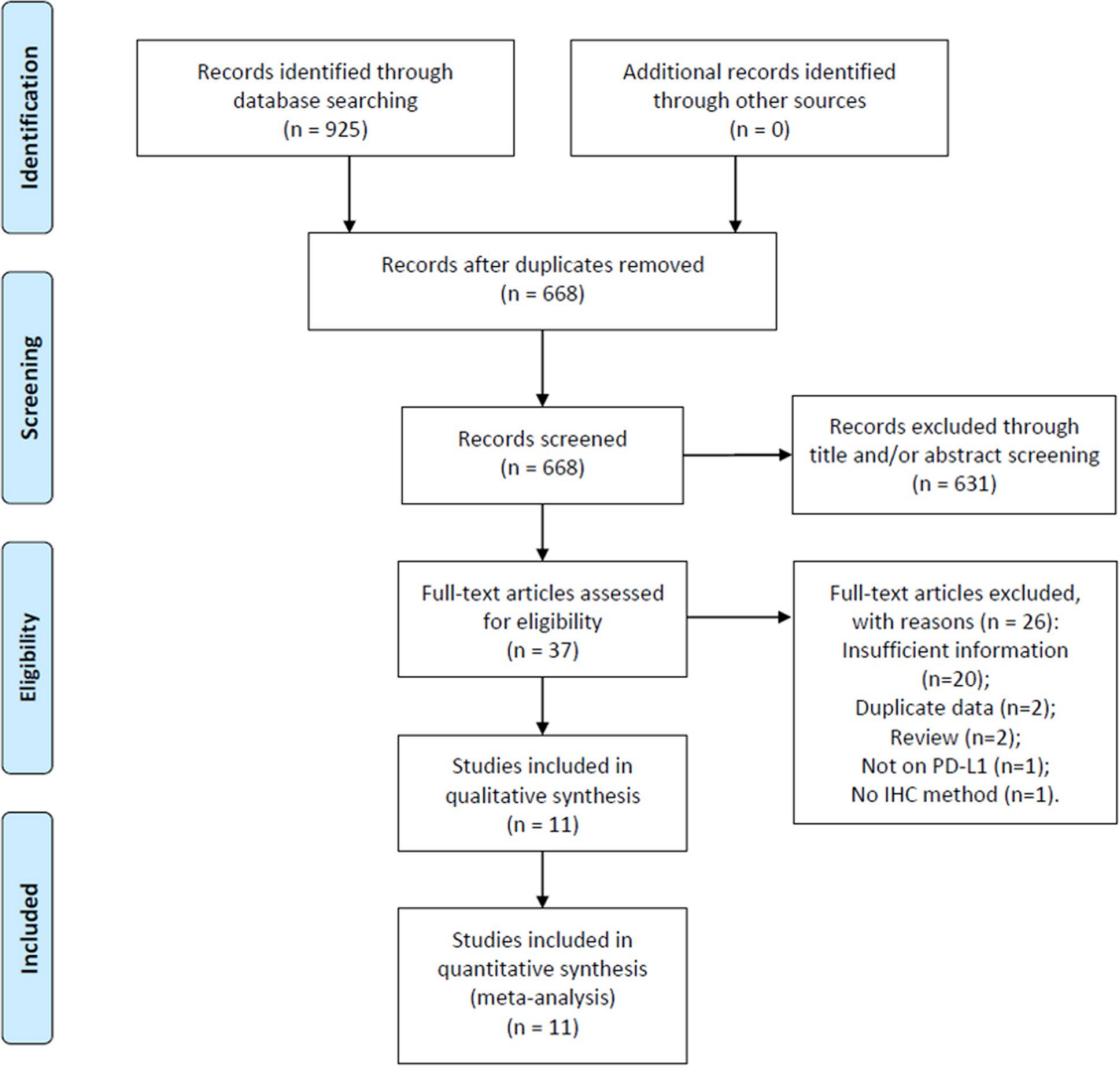
Flowchart for selection of studies.

Initial literature search identified 925 records. After removal of duplicate records, 668 studies remained for further evaluation. Then, 631 recorded were excluded by scanning title and/or abstract. Thirty-seven studies were screened by full-text examination, and 26 studies were excluded for following reasons: 20 studies did not provide sufficient for analysis, 2 studies recruited overlapped patients, 2 studies were reviews, 1 study did not focus on PD-L1, and 1 study did not use IHC method for PD-L1 detection. Ultimately, 11 studies (Nakanishi et al., 2007; Boorjian et al., 2008; Wang et al., 2009; Xylinas et al., 2014; Bellmunt et al., 2015; Wu et al., 2016; Noro et al., 2017; Li et al., 2018b; Pichler et al., 2018; Owyong et al., 2019; Wang et al., 2019) were included in this meta-analysis. The flow diagram is shown in **Figure 1**.

### Study Characteristics

The main characteristics of eligible articles are listed in **Table 1**. The studies were published from 2007 to 2019. Three studies (Wang et al., 2009; Li et al., 2018b; Wang et al., 2019) were conducted in China, three were performed in United States (Boorjian et al., 2008; Xylinas et al., 2014; Bellmunt et al., 2015), two were in Japan (Nakanishi et al., 2007; Noro et al., 2017), and one each in Taiwan (Wu et al., 2016), Austria (Pichler et al., 2018) and Egypt (Owyong et al., 2019). The total sample size was 1,697, ranging from 50 to 318. All studies were a retrospective study design. Regarding clinical outcomes, eight studies reported clinicopathological factors (Boorjian et al., 2008; Wang et al., 2009; Xylinas et al., 2014; Bellmunt et al., 2015; Wu et al., 2016; Li et al., 2018b; Owyong et al., 2019; Wang et al., 2019), eight studies reported OS (Nakanishi et al., 2007; Boorjian et al., 2008; Wang et al., 2009; Xylinas et al., 2014; Bellmunt et al., 2015; Wu et al., 2016; Li et al., 2018b; Wang et al., 2019), five studies described RFS (Nakanishi et al., 2007; Xylinas et al., 2014; Pichler et al., 2018; Owyong et al., 2019; Wang et al., 2019), five studies reported CSS (Nakanishi et al., 2007; Boorjian et al., 2008; Xylinas et al., 2014; Noro et al., 2017; Owyong et al., 2019), and three studies presented DFS (Boorjian et al., 2008; Wu et al., 2016; Noro et al., 2017). Furthermore, all studies were with NOS score ≥ 6, indicating that the studies were of high quality.

**Table 1:** Basic characteristics of included studies.

Author	Year	Country/region	Study design	Duration	No. of patients	Sex (M/F)	Age	Survival analysis	Detection method	NOS score
Nakanishi	2007	Japan	Retrospective	1996–2005	65	47/18	NA	OS, CSS, RFS	IHC	6
Boorjian	2008	USA	Retrospective	1990–1994	318	259/59	69 (37–90)	OS, CSS, DFS	IHC	7
Wang	2009	China	Retrospective	2000–2002	50	40/10	61.7 (42–78)	OS	IHC	7
Xylinas	2014	USA	Retrospective	1988–2003	302	244/58	65.9	OS, CSS, RFS	IHC	8
Bellmunt	2015	USA	Retrospective	NA	160	NA	NA	OS	IHC	6
Wu	2016	Taiwan	Retrospective	NA	120	NA	NA	OS, DFS	IHC	6
Noro	2017	Japan	Retrospective	2004–2014	102	82/20	60 (43–84)	CSS, DFS	IHC	8
Li	2018	China	Retrospective	2009–2011	98	76/22	NA	OS	IHC	7
Pichler	2018	Austria	Retrospective	2006–2015	83	62/21	69 (36–87)	RFS	IHC	8
Owyong	2019	Egypt	Retrospective	1997–2004	151	98/53	52 (36–74)	CSS, RFS	IHC	8
Wang	2019	China	Retrospective	2006–2012	248	214/34	63 (14–94)	OS, RFS	IHC	7

NA, not available; OS, overall survival; CSS, cancer-specific survival; DFS, disease-free survival; RFS, recurrence-free survival; IHC, immunohistochemical staining; NOS, Newcastle-Ottawa Scale.

### Impact of PD-L1 on OS, RFS, CSS, and DFS

**Figure 2:**
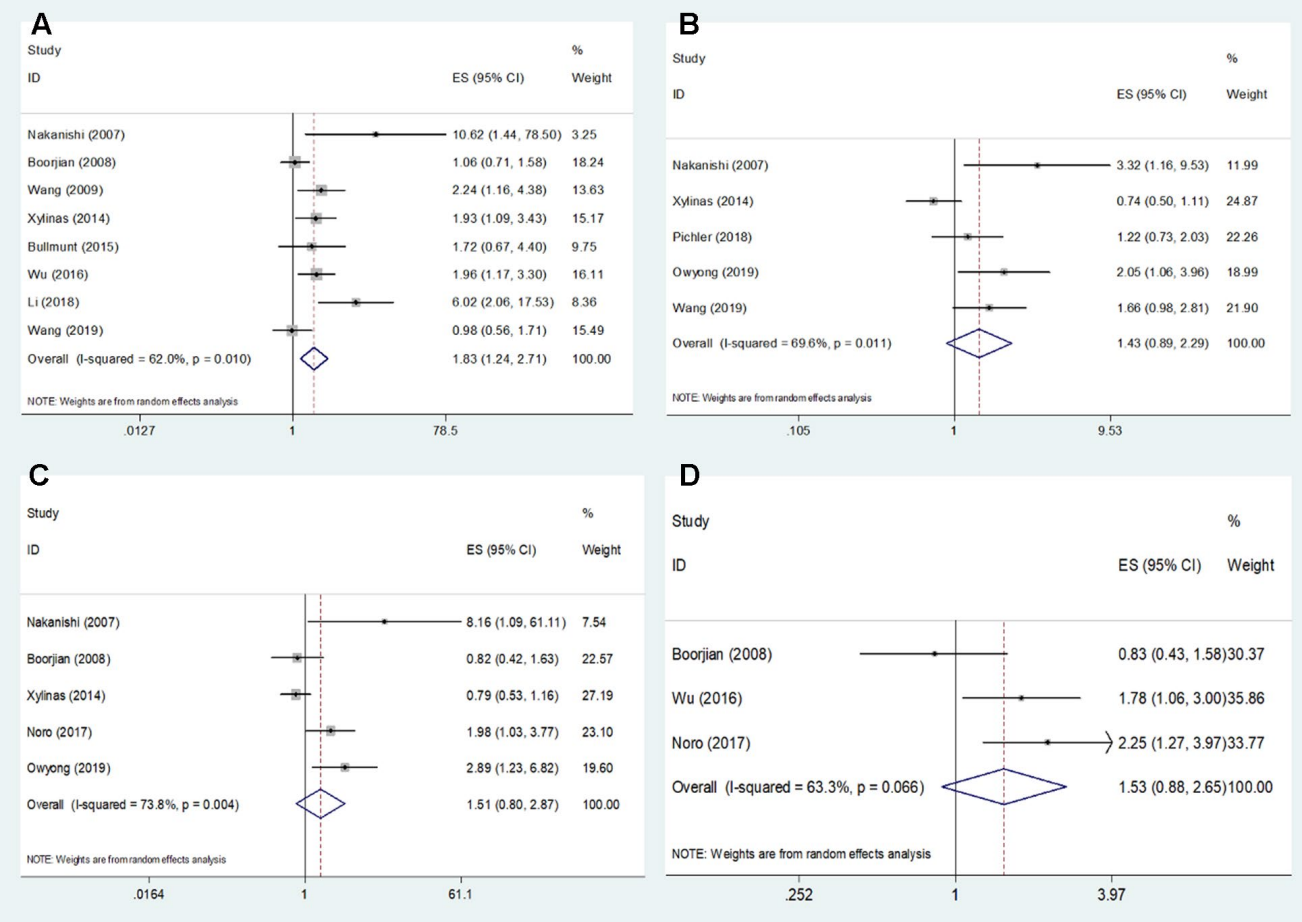
Forest plots describing the association between PD-L1 expression and **(A)** OS, **(B)** RFS, **(C)** CSS, and **(D)** DFS of patients with bladder cancer.

Eight studies (Nakanishi et al., 2007; Boorjian et al., 2008; Wang et al., 2009; Xylinas et al., 2014; Bellmunt et al., 2015; Wu et al., 2016; Li et al., 2018b; Wang et al., 2019) reported data on PD-L1 and OS in bladder cancer. As shown in **Figure 2** and **Table 2**, high PD-L1 was associated with poorer OS (HR = 1.83, 95% CI = 1.24–2.71, *p* = 0.002). Because of significant heterogeneity (*I* ^2^ = 62%, *p* = 0.01), a random-effects model was applied. Five studies (Boorjian et al., 2008; Xylinas et al., 2014; Pichler et al., 2018; Owyong et al., 2019; Wang et al., 2019) showed the relationship between PD-L1 and RFS. The pooled results were HR = 1.43, 95% CI = 0.89–2.29, *p* = 0.134, with significant heterogeneity (*I* ^2^ = 69.6%, *p* = 0.011) (**Table 2**, **Figure 2**). The pooled data from five studies (Nakanishi et al., 2007; Boorjian et al., 2008; Xylinas et al., 2014; Noro et al., 2017; Owyong et al., 2019) suggested nonsignificant association between PD-L1 and CSS in bladder cancer (HR = 1.51, 95% CI = 0.80–2.87, *p* = 0.203; *I* ^2^ = 73.8%, *p* = 0.004, **Table 2**, **Figure 2**). Moreover, three studies reported the correlation of PD-L1 and DFS (Boorjian et al., 2008; Wu et al., 2016; Noro et al., 2017). The random-effects model was applied because there was significant heterogeneity (*I* ^2^ = 63.3%, *p* = 0.066) across the studies. The pooled HR and 95%CI were HR = 1.53, 95% CI = 0.88–2.65, *p* = 0.013 (**Table 2**, **Figure 2**), suggesting PD-L1 was not correlated to worse DFS.

**Table 2:** Meta-analysis of PD-L1 and prognosis in bladder cancer.

Survival analysis	No. of studies	No. of patients	Effects model	HR (95% CI)	p	Heterogeneity	
						*I* ^2^ (%)	p
OS	8	1,361	Random	1.83 (1.24–2.71)	0.002	62	0.01
RFS	5	849	Random	1.43 (0.89–2.29)	0.134	69.6	0.011
CSS	5	938	Random	1.51 (0.80–2.87)	0.203	73.8	0.004
DFS	3	540	Random	1.53 (0.88–2.65)	0.13	63.3	0.066

### PD-L1 and Clinicopathological Features

Eight studies (Boorjian et al., 2008; Wang et al., 2009; Xylinas et al., 2014; Bellmunt et al., 2015; Wu et al., 2016; Li et al., 2018b; Owyong et al., 2019; Wang et al., 2019) explored the association between PD-L1 and clinicopathological characteristics. The pooled data demonstrated that high PD-L1 was significantly correlated with higher tumor stage (OR = 3.9, 95% CI = 2.71–5.61, *p* < 0.001) and distant metastasis (OR = 2.5, 95% CI = 1.22–5.1, *p* = 0.012). However, PD-L1 overexpression was not correlated with other clinicopathological factors including sex (OR = 0.88, 95% CI = 0.65–1.21, *p* = 0.433), tumor grade (OR = 1.19, 95% CI = 0.46–3.09, *p* = 0.72), lymph node status (OR = 1.16, 95% CI = 0.63–2.15, *p* = 0.631), and multifocality (OR = 0.77, 95% CI = 0.5–1.18, *p* = 0.226). The correlation between PD-L1 and clinicopathological parameters is presented in **Table 3**.

**Table 3:** Association of PD-L1 and clinical factors in bladder cancer.

Clinical factors	No. of studies	No. of patients	Effects model	OR (95% CI)	p	Heterogeneity	
						*I* ^2^ (%)	p
Tumor stage (T2–T4 vs Ta–T1)	8	1,447	Fixed	3.9 (2.71–5.61)	< 0.001	0	0.733
Sex (male vs female)	7	1,287	Fixed	0.88 (0.65–1.21)	0.433	13.8	0.325
Tumor grade (high vs low)	6	969	Random	1.19 (0.46–3.09)	0.72	86.5	<0.001
Lymph node status (positive vs negative)	5	1,139	Random	1.16 (0.63–2.15)	0.631	71.7	0.001
Multifocality (multifocal vs unifocal)	4	799	Fixed	0.77 (0.5–1.18)	0.226	0	0.659
Metastasis status (M1 vs M0)	3	466	Fixed	2.5 (1.22–5.1)	0.012	0	0.842

### Publication Bias

**Figure 3:**
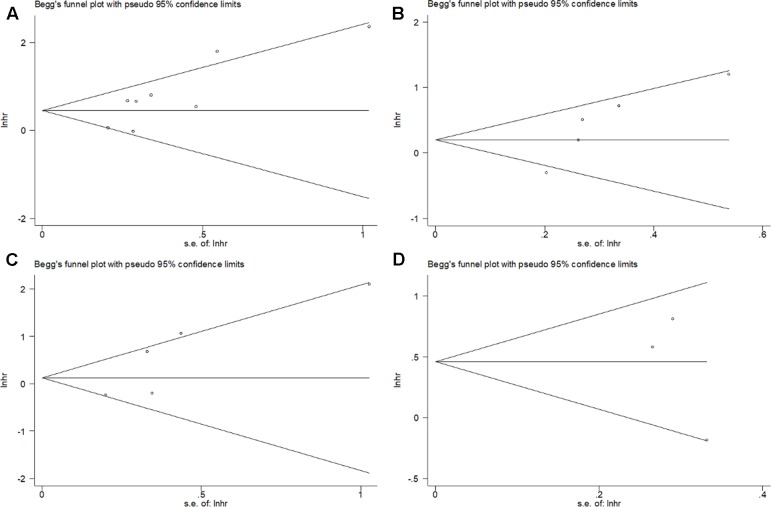
Begg’s funnel plot for publication bias test including PD-L1 expression and **(A)** OS, **(B)** RFS, **(C)** CSS, and **(D)** DFS in bladder cancer patients.

The assessment of the publication bias was carried out by using Begg’s funnel plot test. Begg’s *p* values for OS, RFS, CSS, and DFS were 0.063, 0.086, 0.221, and 0.602, respectively. Begg’s funnel plot was found to be symmetrical (**Figure 3**), indicating no significant publication bias in this meta-analysis.

## Discussion

In the present study, we collected information from 11 recent studies with 1,697 patients and combined the data. The results showed that elevated PD-L1 expression was associated with poorer OS. In addition, PD-L1 overexpression was also connected with higher tumor stage and distant metastasis. There was no obvious evidence of publication bias. The results suggested that PD-L1 expression may be associated with tumor progression and metastasis and could be used as a potential prognostic biomarker. To the best of our knowledge, this is the first pointed meta-analysis investigating the prognostic value of PD-L1 in patients with bladder cancer.

PD-1 and its ligands, PD-L1 and PD-L2, overexpressed in the tumor microenvironment (Riley, 2009). The interaction of PD-1/PD-L1 can inhibit T-cell activation and proliferation, cytokine production, and cytolytic function (Riley, 2009). In addition, PD-L1 can also stimulate IL-10 production in T cells to mediate immune suppression (Dong et al., 1999). PD-L1 was found to be overexpressed in multiple solid tumor types to generate an immunosuppressive tumor microenvironment (Iwai et al., 2002; Blank et al., 2005; Wang et al., 2017). In the present study, we found the association of PD-L1 and higher tumor stage and distant metastasis, which implied the role of PD-L1 in tumor development. A recent study showed that PD-L1 played a critical role in promoting epithelial-to-mesenchymal transition (EMT) phenotype of esophageal cancer (Chen et al., 2017). Another study also suggested that PD-L1 expression was a significant risk factor for nodal metastasis in cutaneous squamous cell carcinoma (Garcia-Pedrero et al., 2017). The activation of IL-6/STAT3/PD-L1 pathway was found to be involved in the EMT process in bladder cancer (Zhang et al., 2019).

A number of previous studies also reported the prognostic significance of PD-L1 in various cancers. A recent meta-analysis including 2,005 patients showed that high PD-L1 expression was associated with a poor prognosis (HR = 2.04, 95% CI = 1.18–3.54, *p* = 0.01) in non-Hodgkin lymphoma (Zhao et al., 2018). Li’s study showed that PD-L1 overexpression could foresee worse OS and DFS in hepatocellular carcinoma (Li et al., 2018a). In addition, another meta-analysis comprising a total of nine studies with 993 patients demonstrated that elevated PD-L1 expression was related with poor OS (HR = 1.63, 95% CI = 1.34–1.98, *p* < .001) and CSS (HR = 1.86, 95% CI = 1.34–2.57, *p* < .001) in pancreatic cancer (Hu et al., 2019). High PD-L1 expression was also correlated with poor OS in breast cancer (Zhang et al., 2017). The results of our study were in line with previous studies, suggesting the prognostic value of PD-L1 in bladder cancer. Furthermore, we also found the connection between PD-L1 and distant metastasis in bladder cancer, which may be explained by the role of PD-L1 in EMT process (Zhang et al., 2019). Recently, many studies also reported the effectiveness and patient-reported outcomes in clinical trials of PD-L1 inhibitors. Madore et al. showed that PD-L1 expression in melanoma showed marked heterogeneity within and between patients, which supported the therapeutic strategies of melanoma patients in a PD-L1-based manner (Madore et al., 2015). In addition, stage III melanoma patients with negative PD-L1 expression is associated with worse survival and immune response (Madore et al., 2016). A recent meta-analysis demonstrated that PD-L1 expression was significantly associated with mortality and clinical response to anti-PD-1/PD-L1 antibodies in metastatic melanoma patients (Gandini et al., 2016). The health-related quality of life was also better in advanced cancer patients receiving PD-1/PD-L1 inhibitors than in those receiving standard-of-care therapy (Nishijima et al., 2019). Those studies suggest that the clinical management of PD-1/PD-L1 inhibitors is complex and should be adjusted in the individual patient level.

Notably, age is also a risk factor for bladder cancer patients. In the included studies, five studies (Xylinas et al., 2014; Wu et al., 2016; Li et al., 2018b; Owyong et al., 2019; Wang et al., 2019) provided the data on age in PD-L1 (+) and PD-L1 (−) groups. However, three studies (Xylinas et al., 2014; Wu et al., 2016; Owyong et al., 2019) presented age in the format of median (range). One study (Li et al., 2018b) reported the number of patients in PD-L1 (+) and PD-L1 (−) groups using 65 years as threshold. One study used 60 years (Wang et al., 2019) to divide patients. Therefore, the quantitative analysis of PD-L1 expression and age could not be performed because of different cutoff values of age (65 and 60 years). In spite of this, we can find that patients with PD-L1 (+) expression are older than patients with PD-L1 (−) expression in four studies (Xylinas et al., 2014; Wu et al., 2016; Li et al., 2018b; Wang et al., 2019). All five studies (Xylinas et al., 2014; Wu et al., 2016; Li et al., 2018b; Owyong et al., 2019; Wang et al., 2019) reported nonsignificant association between age and PD-L1 expression (all *p* > 0.05). Moreover, in the analysis of association between PD-L1 expression and clinical factors, heterogeneity was found on sex, tumor grade, and lymph node status (**Table 3**). Because different studies may select patients with various criteria, the heterogeneity among studies may be inherent and may exist. In this occasion, we applied different effects model according to different heterogeneity.

Some limitations need to be mentioned in this meta-analysis. First, the determination of high expression of PD-L1 might vary in the studies because of different cutoff values, which may introduce potential bias. Second, the sample size was relatively small. Only 11 studies with 1,697 patients were included for analysis. For example, for CSS and DFS analysis, only five and three studies were included; the small study may compromise the credibility of the results. Third, although we did not find publication bias in the meta-analysis, the publication bias and selection bias could possibly exist. As we know, studies with significant results are inclined to be published (Koletsi et al., 2009). Therefore, the results should be treated with caution.

## Conclusion

In summary, the findings of this meta-analysis suggest that elevated PD-L1 expression is associated with poor survival, higher tumor stage, and distant metastasis in bladder cancer. PD-L1 may be useful in the future as a novel prognostic factor in bladder cancer. Nevertheless, due to some limitations, well-designed, multicenter randomized controlled trials should be performed.

## Data Availability

All datasets generated for this study are included in the manuscript/supplementary files.

## Author Contributions

LZ, JS, and ZBL designed the study. LZ, JS, LiW, ZGL, and LeW performed the research. LZ and JS collected and analyzed the data. LZ and JS wrote the paper. LeW amended the article. ZBL acts as the submission’s guarantor and takes responsibility for the integrity of the work as a whole, from inception to published article. All authors reviewed the manuscript. All authors read and approved the final manuscript.

## Conflict of Interest Statement

The authors declare that the research was conducted in the absence of any commercial or financial relationships that could be construed as a potential conflict of interest.
